# Integration and exchange of split dCas9 domains for transcriptional controls in mammalian cells

**DOI:** 10.1038/ncomms13056

**Published:** 2016-10-03

**Authors:** Dacheng Ma, Shuguang Peng, Zhen Xie

**Affiliations:** 1 MOE Key Laboratory of Bioinformatics and Bioinformatics Division, Center for Synthetic and System Biology, Department of Automation, Tsinghua National Lab for Information Science and Technology, Tsinghua University, Beijing 100084, China

## Abstract

Programmable and precise regulation of dCas9 functions in response to multiple molecular signals by using synthetic gene circuits will expand the application of the CRISPR-Cas technology. However, the application of CRISPR-Cas therapeutic circuits is still challenging due to the restrictive cargo size of existing viral delivery vehicles. Here, we construct logic AND circuits by integrating multiple split dCas9 domains, which is useful to reduce the size of synthetic circuits. In addition, we engineer sensory switches by exchanging split dCas9 domains, allowing differential regulations on one gene, or activating two different genes in response to cell-type specific microRNAs. Therefore, we provide a valuable split-dCas9 toolkit to engineer complex transcription controls, which may inspire new biomedical applications.

## Abstract (web summary)

Molecular engineering of Cas9 has the potential to expand the application of CRISPR-Cas technology. Here, Ma *et al*. show that dCas9 can be split and reconstituted in human cells and use a domain swapping strategy to engineer custom Cas9-based logic circuits and sensory switches.

## Graphical abstract

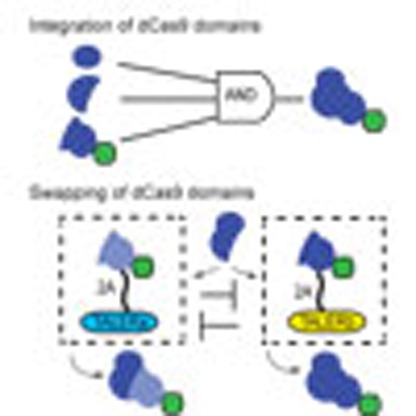

The CRISPR-associated protein 9 (Cas9) discovered from *Streptococcus pyogenes* is a multi-domain protein, which has been widely used in genome editing and transcriptional control in mammalian cells due to its superior modularity and versatility^1^,^2^,^3^,^4^. The Cas9-DNA targeting specificity is determined by both the Cas9-associated guide RNA (gRNA) and a short protospacer adjacent motif (PAM) directly downstream of the DNA recognition site^5^,^6^. The *S. pyogenes* Cas9 (SpCas9) protein usually consists of a recognition lobe and a nuclease lobe. The recognition lobe contains a bridge helix (residues 60–93), a REC1 (residues 94–179 and 308–713) domain and a REC2 (residues 180–307) domain, while the nuclease lobe includes a RuvC (residues 1–59, 718–769 and 909–1,098) domain, a HNH (residues 775–908) domain and a PAM-interacting (PI) (residues 1,099–1,368) domain^7^. Structural analysis of the SpCas9:DNA:gRNA complex has facilitated the engineering of mutant SpCas9 proteins that recognize variant PAM sequences^8^,^9^. For example, D1135E/R1335Q/T1337R mutations (EQR) or D1135V/G1218R/R1335E/T1337R mutations (VRER) in the PI domain can switch the PAM specificity of SpCas9 from NGG to NGCG (ref. 9). Furthermore, the functional Cas9 protein can be reconstituted from two inactive split-Cas9 peptides in the presence of gRNA (ref. 10), by using a split-intein protein splicing strategy^11^,^12^, or by respectively fusing to dipartite domains that interact with each other^13^,^14^,^15^,^16^. Inteins often require cysteine, serine or threonine at the +1 amino acid position immediately downstream of the C-terminal intein fragment to complete the self-catalytic splicing reaction^12^. In the split-intein protein splicing system, the split Cas9 fragments are fused to either a N-terminal intein fragment or a C-terminal intein fragment, which can associate with each other and catalytically splice the two split Cas9 fragments into one Cas9 protein^11^,^12^.

Several strategies have been developed to engineer modular and layered gene circuits in mammalian cells by regulating dCas9 and gRNA expression^17^,^18^,^19^,^20^. Transcriptional controls in mammalian cells can be achieved by directly fusing a transcriptional regulatory domain to the nuclease deactivated Cas9 (dCas9) (refs 1, 4). Alternatively, multiple transcriptional regulatory domains can be recruited to the dCas9 by tagging the dCas9 with a repeating peptide scaffold, or by fusing repeating RNA motifs to the cognate gRNA (refs 21, 22). However, biomedical applications of the CRISPR-Cas system demand the exploration of new platforms for engineering mammalian synthetic circuits that integrate and process multiple endogenous inputs. In addition, the application of CRISPR-Cas therapeutic circuits is also challenging due to the restrictive cargo size of existing viral delivery vehicles. Alternatively, the split Cas9 system can be used to bypass the packing limit of the viral delivery vehicles^11^.

In this study, we verify dCas9 can be split and reconstituted in human cells. Then we engineer logic AND circuits and sensory switches by integrating and swapping split-dCas9 domains, which may reduce the size of synthetic circuits comparing to the circuits that use the full-length dCas9. We further connect small molecule, shRNA or cell-type specific miRNA inputs to control these Cas9-based synthetic circuits. Such a platform may inspire new biomedical applications by using the CRISPR-Cas system.

## Results

### Functional reconstitution of split Cas9 domains

**Figure 1:**
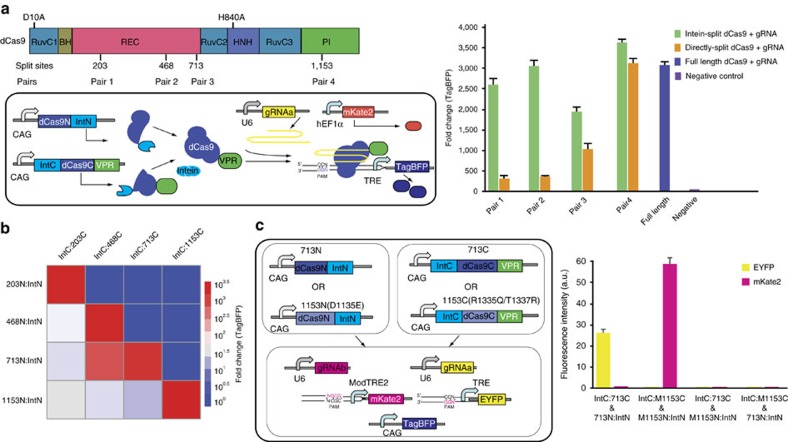
Characterization of split dCas9 domains. (**a**) Diagram of dCas9 domain organization and split sites (top left), and reconstitution of split dCas9 domains for transcription activation (lower left). The mKate2 was used as an internal control. The TRE promoter contains seven repeats of gRNAa binding sites with NGG PAM sequences. (**b**) Orthogonality of split dCas9 domains. (**c**) Improved orthogonality of split dCas9 domains by introducing mutations in the PAM recognition domain. The ModTRE2 promoter contains seven repeats of gRNAb binding sites with NGCG PAM sequences. (**a**,**c**) Each bar shows mean fold change (mean±s.e.m.; *n*=3) of indicated fluorescence measured by using flow cytometer 48 h after transfection.

According to the Cas9 sequence and structural information, as well as previous reports^8^,^13^, we selected four potential split sites where serine is at the +1 amino acid position when fused to the C-terminal Intein fragment^12^. All four potential split sites were surface residues and located in the loop region, which may be more accessible for intein trans-splicing reaction and have less effect on the protein folding^8^. Then, we constructed eight pairs of split Cas9 constituents that either fused to the N-terminal (IntN) and C-terminal (IntC) split inteins or not (Fig. 1a and Supplementary Fig. 1a). As shown in Supplementary Fig. 1b, we inserted a repeat sequence in the middle of enhanced yellow fluorescent protein (EYFP) reporter gene as described^23^. The functional Cas9 protein can cleave the EYFP repeat region, triggering the reconstitution of inactive EYFP into the full-length active EYFP reporter gene. By using this EYFP-reconstitution reporter system, we found that all four intein-mediated split-Cas9 pairs efficiently reactivated the EYFP expression in a human embryonic kidney HEK293 cells (Supplementary Fig. 1c). The Cas9 sets split at residues 203, 468, 713 and 1,153 without intein fusion, displayed a reduced activity compared with their counterparts with appropriate intein fusions (Supplementary Fig. 1c). Next, we tested whether a similar set of split dCas9:VPR pairs can recapitulate the function of the full-length dCas9:VPR in HEK293 cells by a transient transfection experiment. Three of split pairs without intein fusions showed a reduced activation function compared with their counterparts with intein fusions (Fig. 1a). In contrast, the dCas9 protein directly split at position 1,135 was almost as active as the intact dCas9 protein (Fig. 1a). Three of the split dCas9:VPR pairs fused to intein fragments activated the reporter gene as efficiently as the full-length dCas9:VPR, while the dCas9:VPR split at residue 713 was not as efficient (Fig. 1a). These results suggest that the VPR fusion and the choice of split site might affect reconstitution of split dCas9 fragments and then influence the protein activity. We further demonstrated the functional reassembly of dCas9 constituents split at either residue 713 or residue 1,153 when fused to different transcription regulatory domains, such as Krab, Suntag and VP64 (Supplementary Fig. 2).

Next, we explored whether split dCas9 fragments across different split pairs can be reconstituted into a functional dCas9. The experiment demonstrated that combinations of dCas9 IntN and IntC fragments that resulted in incomplete dCas9 proteins failed to activate TagBFP expression (Fig. 1b). In contrast, fragment combinations that provided the complete polypeptide sequence activated TagBFP expression even when the two fragments were partly redundant. Interestingly, the dCas9 pair split at residue 1,153 divided the PI domain into two fragments. We then tested whether it is possible to increase the orthogonality of the split set at residue 1,153 by introducing EQR mutations in the PI domain^9^. As shown in Fig. 1c, the reconstitution of the split dCas9 pair at residue 1,153 with the EQR mutations only activated the mKate2 reporter gene with the NGCG PAM but not the EYFP reporter gene with the NGG PAM, while the reconstitution of the split dCas9 pair at residue 713 without mutations led to the opposite results. In addition, no cross activity was observed when either the wild-type N-terminal or C-terminal dCas9 fragment was combined with the EQR mutant C-terminal or N-terminal dCas9 constituents (Fig. 1c). These orthogonal split dCas9 pairs would potentially facilitate the construction of complex genetic circuits and logic gates.

### Construction of three-input logic AND circuit

**Figure 2:**
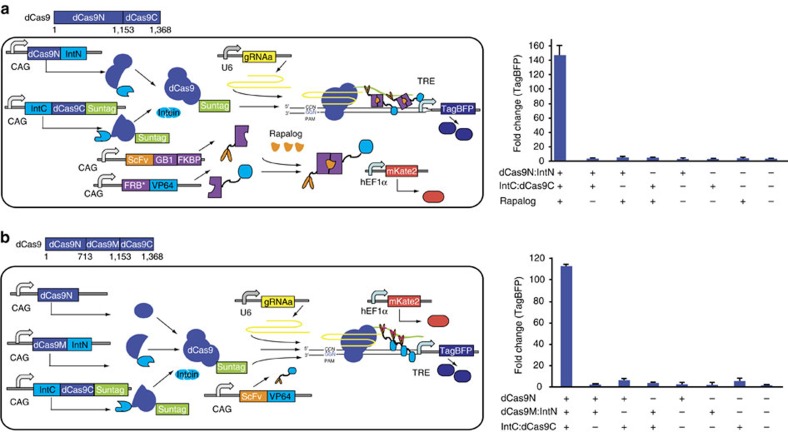
Construction of logic AND circuits by integrating split dCas9 domains. (**a**) Schematic representation of a three-input logic AND circuit by using two split dCas9 fragments (dCas9N:IntN and IntC:dCas9C:Suntag) and the rapalog that promote the dimerization of ScFv:GB1:FKBP and FRB*:VP64. The dCas9 constituents are split at residue 1153. (**b**) Schematic representation of reconstitution of three split-dCas9 fragments. The dCas9 constituents are split at residue 713 and 1153. (**a**,**b**) Data are shown as the mean fold change (mean±s.e.m.; *n*=3) of TagBFP fluorescence measured by using flow cytometer 48 h after transfection into HEK293 cells.

On the basis of the above results, we started to engineer a logic AND circuit by using the dCas9 constituents split at residue 1,153 and the Suntag repetitive peptide scaffold that contains ten ScFv binding motifs (Fig. 2a)^21^. The ScFv along with a small solubility tag GB1 and VP64 fragments were respectively fused to FK506 binding protein 12 (FKBP) and FKBP rapamycin binding (FRB*) domains with a T2089L mutation derived from the mammalian target of rapamycin (mTOR)^21^,^24^. Therefore, the resulted fusion proteins, ScFv:GB1:FKBP and FRB*:VP64 can form a heterodimer in the presence of the rapamycin analogue AP21967 (rapalog). We tested the function of a three-input logic AND circuit that used split-dCas9 constituents and the rapalog in HEK293 cells. The logic AND circuit operated correctly in response to all eight different combinations of three inputs with an ON/OFF ratio greater than 140-fold (Fig. 2a). As shown in Fig. 1, dCas9:VPR pairs split at residues 713 and 1,153 without intein more efficiently activated the expression of TagBFP than the other two split pairs. Therefore, we constructed a three-input logic AND circuit by splitting dCas9 into three fragments based on these results, including dCas9N containing dCas9 residues from 1 to 713, dCas9M:IntN containing the residues from 714 to 1,153, and IntC:dCas9C:Suntag containing the residues from 1,154 to 1,368. As expected, this split-dCas9 logic AND circuit induced TagBFP expression greater than 110-fold only when all three split-dCas9 constituents were added in HEK293 cells (Fig. 2b).

### Two-input and one-output sensory switch

**Figure 3:**
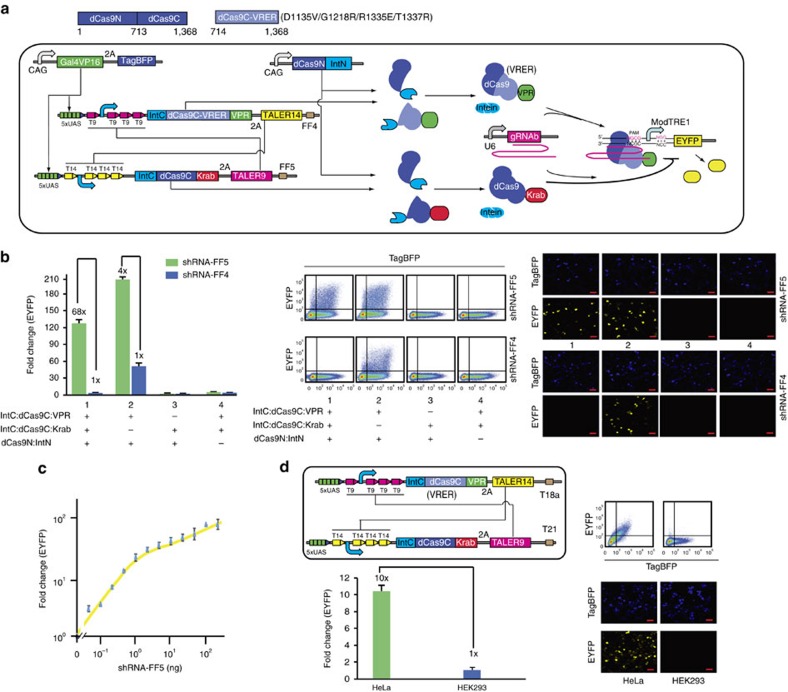
Two-input and one-output sensory switch by swapping split dCas9 domains. (**a**) Schematic representation of sensory switches by exchanging split dCas9 domains through a TALER sensory switch. The dCas9 constituents are split at residue 713. The light blue rectangle represents the mutant dCas9 domain (D1135V/G1218R/R1335E/T1337R or VRER in short) that can recognize the NGCG PAM sequences but not the NGG PAM sequences. The ModTRE1 promoter contains seven repeats of gRNAb binding sites with the NGCG PAM sequences upstream of a miniCMV core, followed by three repeats of gRNAb binding sites with the NGG PAM sequences. (**b**) Setting states of the sensory switch by artificial shRNA-FF5 and shRNA-FF4. Representative flow cytometry scatter plots and microscopic images are shown in the right panel. Each red scale bar in images represents 50 μm. (**c**) Dosage response of the sensory switch to shRNA-FF5. The solid line was plotted by using qplot function in R package. (**d**) Control of sensory switches by indicated endogenous microRNAs in HeLa and HEK293 cells. Schematic representation of sensory switches controlled by endogenous microRNAs. For simplicity, only the core of the sensory switch is shown in the top left panel. Representative flow cytometry scatter plots and microscopic images are shown in the right panel. Each red scale bar in images represents 50 μm. (**b**–**d**) Data shown as the mean fold change (mean±s.e.m.; *n*=3) of EYFP fluorescence was measured 48 h after transfection.

We recently developed a TALER sensory switch controlled by two different shRNAs and microRNAs (ref. 25). To test the domain exchange of dCas9 constituents, we respectively fused IntC:dCas9C:VPR and IntC:dCas9C:Krab to TALER14 and TALER9, which reconstituted with a constitutive dCas9N:IntN to activate or repress the expression of the EYFP reporter gene by competitively binding to the TRE promoter. The VPR activation domain was chosen because the activation efficiency is greater than both VP64 and Suntag activation domains in our experimental setup (Supplementary Fig. 2b). As shown in Supplementary Fig. 3b, the shRNA-FF5 and shRNA-FF4 respectively triggered the ON and OFF states of the sensory switch with a ON/OFF ratio of 51-fold. To further improve the performance of sensory switch, we used IntC:dCas9C-VRER:VPR that contained a mutant PI domain (D1135V/G1218R/R1335E/T1337R) to switch the PAM recognition specificity of the reconstituted dCas9 from NGG to NGCG (Fig. 3a). Accordingly, we constructed a modified TRE promoter (ModTRE1) that contained 7 gRNAb binding sites with the NGCG PAM sequences upstream of the minimal CMV promoter, followed by three gRNAb binding sites with the NGG PAM sequences. The results showed that the ON/OFF ratio of this modified sensory switch increased to 68-fold (Fig. 3b). In contrast, the shRNA-FF4 failed to efficiently repress the EYFP expression in the absence of the feedback regulation exerted by the 2A-linked IntC:dCas9C:Krab and TALER9 (Fig. 3b). Furthermore, the sensory switch responded to the shRNA-FF5 input in a dosage dependent manner (Fig. 3c).

Next, we connected cell-type specific microRNAs to control the sensory switch by fusing four tandem repeats of fully complementary microRNA binding sites in the 3′-UTR of the IntC:dCas9C:VPR-2A-TALER14 and IntC:dCas9C:Krab-2A-TALER9 (Supplementary Fig. 3c). We previously reported that miR18a, miR191, miR19a-3p and miR19b-3p that highly expressed in HEK293 cells but not in HeLa which can be used as the HEK293 specific microRNA markers, while miR21 can be used as the HeLa specific microRNA marker^25^,^26^. After transfection into HeLa and HEK293 cells, the sensory switch responded correctly to miR21/miR18a, miR21/miR191 and miR21/miR19ab (a composite marker that includes both miR19a-3p and miR19b-3p) with a ON/OFF ratio of 7-fold, 3.6-fold and 2.5-fold respectively (Supplementary Fig. 3c). These results suggested that the sensory switch can recapitulate the function of either dCas9:VPR or dCas9:Krab in response to two different endogenous microRNAs. We then optimized the sensory switch circuit by replacing IntC:dCas9C:VPR with IntC:dCas9C-VRER:VPR, increasing the ON/OFF ratio to 10-fold in response to miR21/miR18a input combination (Fig. 3d). In addition, the sensory switch responded to the miR21 input in a dosage dependent manner (Supplementary Fig. 4).

### Two-input and two-output sensory switch

**Figure 4:**
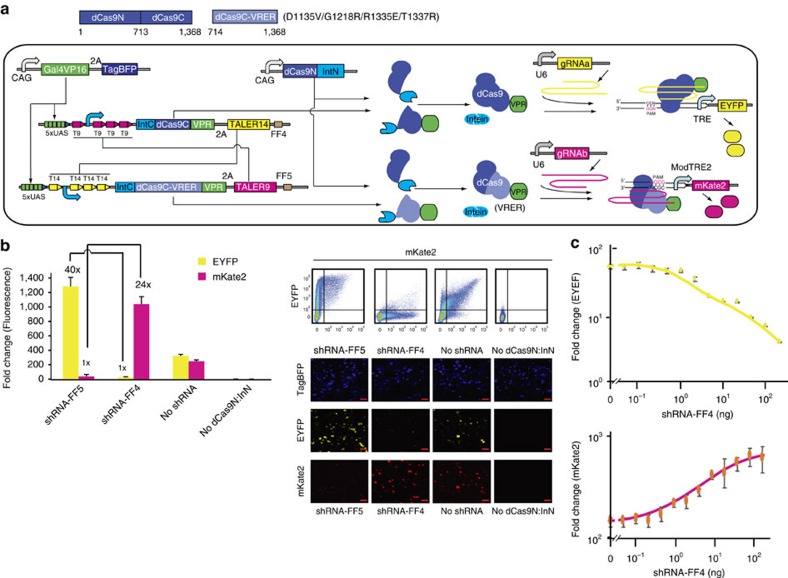
Two-input and two-output sensory switch. (**a**) Schematic representation of a two-input and two-output sensory switch by swapping split dCas9 domains that recognize two different PAM sequences. The dCas9 constituents are split at residue 713. The light blue rectangle represents the mutant dCas9 domain (VRER) that can recognize the NGCG PAM sequences but not the NGG PAM sequences. The ModTRE2 promoter contains seven repeats of gRNAb binding sites with the NGCG PAM sequences upstream of a miniCMV core. (**b**) Setting states of the sensory switch by artificial shRNA-FF5 and shRNA-FF4. Representative scatter plots and microscopic images are shown in the right panel. Each red scale bar in images represents 50 μm. (**c**) The response of the sensory switch to varying amount of shRNA-FF4. The solid line was plotted by qplot function in R package. (**b**,**c**) Each bar or data point shows mean fold changes (mean±s.e.m.; *n*=3) of EYFP or mKate2 measured by using flow cytometer 48 h after transfection.

To test whether the sensory switch can be used to activate two different output genes in response to two different shRNAs, we replaced the IntC:dCas9C:Krab with the orthogonal activator IntC:dCas9C-VRER:VPR (Fig. 4a). The orthogonality test showed that IntC:dCas9C-VRER:VPR only activated the modified TRE promoter (ModTRE2) with the NGCG PAM sequences but not the original TRE promoter with the NGG PAM sequences (Supplementary Fig. 5). As shown in Fig. 4b, the shRNA-FF5 and shRNA-FF4 respectively induced a high level of EYFP and mKate2 with a greater than 20-fold ON/OFF ratio, although we observed a leaky expression of both EYFP and mKate2 at the OFF state. As expected, the EYFP level was gradually decreased when increasing the amount of shRNA-FF4, while the mKate2 level was increased in a shRNA-FF4 dosage dependent manner (Fig. 4c). Because dCas9-VRER:VPR only activated the modified TRE promoter (ModTRE2) with the NGCG PAM sequences but not the original TRE promoter with the NGG PAM sequences (Supplementary Fig. 5), we hypothesize that the leaky expression may be due to the trace of split dCas9 activation domains at the OFF state of the sensory switch. To address this issue, we first demonstrated that applying both a weak transcriptional repression by the dCas9-Krab and a weak post-transcriptional repression by exogenously introducing miR21 can greatly reduce the leaky expression of EYFP, although the EYFP level at the ON state also decreased (Supplementary Fig. 6a,b,d). We previously showed that the feed-forward loop is a useful circuit architecture to reduce expression leakiness^26^. We fused four tandem repeats of shRNA-FF4 target site in the 3′-UTR of EYFP and four tandem repeats of shRNA-FF5 target site in the 3′-UTR of the mKate2. In this setup, shRNA-FF4 and shRNA-FF5 repressed EYFP and mKate2 through a feed-forward loop, respectively. As shown in Supplementary Fig. 6e, almost no leaky expression of either EYFP or mKate2 was observed.

## Discussion

In this study, we demonstrated that split dCas9 domains can be reconstituted for transcriptional regulations in cultured human cells (Fig. 1), allowing modular and efficient construction of three-input logic AND circuits (Fig. 2). By using the split dCas9 and Suntag system, it is possible to easily increase the number of inputs up to seven, including three split dCas9 domains, two Suntag fragments, the rapalog and the gRNA. In addition, we developed an orthogonal split dCas9 pair by introducing mutations in the PI domains^9^, which recognized the NGCG PAM sequences instead of the NGG PAM sequences (Fig. 1c). These orthogonal split dCas9 pairs would be a useful toolkit to construct complex and layered logic gates with multiple inputs. In addition, a similar strategy can be applied to engineering split Cas9 pairs with nuclease or nickase activity.

Recently, several strategies have been developed to control the Cas9/dCas9 activity by using small molecules and light signals^13^,^14^,^15^,^27^,^28^,^29^. Connecting tissue and cellular specific inputs to regulate Cas9/dCas9 activity can facilitate the application of the CRISPR-Cas system in basic and translational biomedical researches^20^,^30^. In this study, we developed sensory switches by exchanging split dCas9 domains, allowing differential regulations on one gene, or activating two different genes in response to cell-type specific microRNAs (Figs 3 and 4). We anticipate that combining our sensory switch with other tissue and cellular inputs will inspire new approaches for more complex regulations on the Cas9/dCas9 function.

It has been shown that split Cas9 system can be delivered *in vivo* by using recombinant adenovirus-associate viruses (rAAV)^11^,^12^. Our circuit design principles provided an useful method to reduce the size of synthetic circuits by integrating and swapping split Cas9/dCas9 domains fused with different functional domains. It will be interesting to adapt our split Cas9/dCas9 system to rAAV delivery system, and test the Cas9/dCas9 activity to edit and regulate endogenous genes *in vivo*. Such a CRISPR-Cas9 system will be particularly appealing in biomedical applications in which viral delivery vehicles with a restrictive cargo size are preferred.

## Methods

### Reagents and enzymes

Restriction endonuclease, polynucleotide kinase (PNK), T4 DNA ligase, Quick DNA ligase and Q5 High-Fidelity DNA Polymerase were purchased from New England Biolabs. Oligonucleotides were synthesized by Genewiz and Sangon Biotech. Gateway LR reaction (Life Technologies) was performed by following the manufacturer's protocol. The guide sequences of gRNAs are listed in Supplementary Table 1.

### Plasmid DNA constructs

The methods used for plasmid DNA construction are summarized in Supplementary Information. The oligonucleotide primer sequences (purchased from Genewiz Inc.) are listed in Supplementary Table 2. The protein and DNA sequences of plasmid DNA constructs are listed in Supplementary Data 1. Diagrams of the TRE and derivative promoters (ModTRE1 and ModTRE2) are shown in Supplementary Fig. 7.

### Cell culture and transfection

HEK293 (293-H) cell line was purchased from Life Technologies. Hela S3 cell line was purchased from ATCC. HEK293 or HeLa S3 cells were cultured in high-glucose DMEM complete media (Dulbecco's modified Eagle's medium (DMEM), 4.5 g l^−1^ glucose, 0.045 units ml^−1^ of penicillin, 0.045 g ml^−1^ streptomycin, and 10% FBS (Life Technologies)) at 37 °C, 100% humidity, and 5% CO_2_.

One day before transfection, ∼1.2 × 10^5^ HEK293 cells in 0.5 ml of high-glucose DMEM complete media were seeded into each well of 24-well plastic plates (Falcon). Shortly before transfection, the medium was replaced with fresh DMEM complete media. The transfection experiments were performed by using either Lipofectamine LTX (Life Technologies) or Attractene transfection reagent (Qiagen) by following the manufacturer's protocol. The amount of plasmid DNAs used in transfection experiments is listed in Supplementary Table 3. Cells were cultured for 2 days before flow cytometry analysis.

### Flow cytometry

Cells were trypsinized 48 h after transfection and centrifuged at 300*g* for 7 min at 4 °C. The supernatant was removed, and the cells were resuspended in 1 × PBS that did not contain calcium or magnesium. Fortessa flow analyser (BD Biosciences) was used for fluorescence-activated cell sorting analysis with the following settings. TagBFP was measured using a 405 nm laser and a 450/50 filter with a photomultiplier tube (PMT) set at 275 V. EYFP was measured with a 488 nm laser and a 530/30 filter using a PMT set at 240 V. mKate2 was measured with a 561 nm laser and a 670/30 filter using a PMT set at 450 V. For each sample, ∼1 × 10^4^ to ∼5 × 10^4^ cell events were collected.

### Fluorescence microscopy

The cells were grown on class bottom cell culture dish (ϕ 20 nm) in complete media for transfection. Approximately 48 h after transfection, fluorescent images of cultured cells were captured by Zeiss LSM780. The filter sets (Chroma) are as follows. TagBFP fluorescence was measured with an excitation at 405 nm and an emission in the range of 410–507 nm. EYFP fluorescence was measured with an excitation at 514 nm and an emission in the range of 519–568 nm. mKate2 fluorescence was measured with an excitation at 543 nm and an emission in the range of 571–651 nm. Image acquisition and post-acquisition analysis were performed using ZEN 2011.

### Data availability

The authors declare that all data supporting the findings of this study are available within the article and its Supplementary Information files or are available from the corresponding author upon request.

## Additional information

**How to cite this article:** Ma, D. *et al*. Integration and exchange of split dCas9 domains for transcriptional controls in mammalian cells. *Nat. Commun.* **7,** 13056 doi: 10.1038/ncomms13056 (2016).

## Supplementary Material

Supplementary Information Supplementary Figures 1-7, Supplementary Tables 1-3, Supplementary Methods and Supplementary References

Supplementary Data 1 The protein and DNA sequences of plasmid DNA constructs
