# Supplementary material for: Integration and exchange of split dCas9 domains for transcriptional controls in mammalian cells
Source: Nat Commun. 2016 Oct 3;7:13056. doi: 10.1038/ncomms13056 (PMC5063958; doi:10.1038/ncomms13056)
Supplement: Supplementary Information — Supplementary Figures 1-7, Supplementary Tables 1-3, Supplementary Methods and Supplementary References [file ncomms13056-s1.pdf]

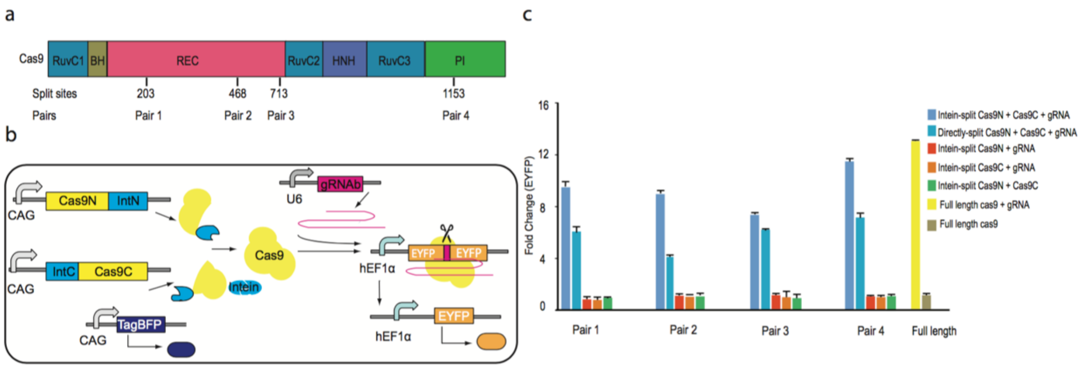


**Supplementary Figure 1 | Integration of split Cas9 constituents for gene editing**

**a.** Diagram of Cas9 domain organization and split sites. **b.** Diagram of reconstitution of split Cas9 domains for gene editing. **c.** Gene editing efficiency by integration of split Cas9 domains with or without split-intein fusions. Each bar shows mean fold changes (mean ± SEM; n = 3) of EYFP fluorescence measured by using flow cytometer 48 h after transfection in HEK293 cells.

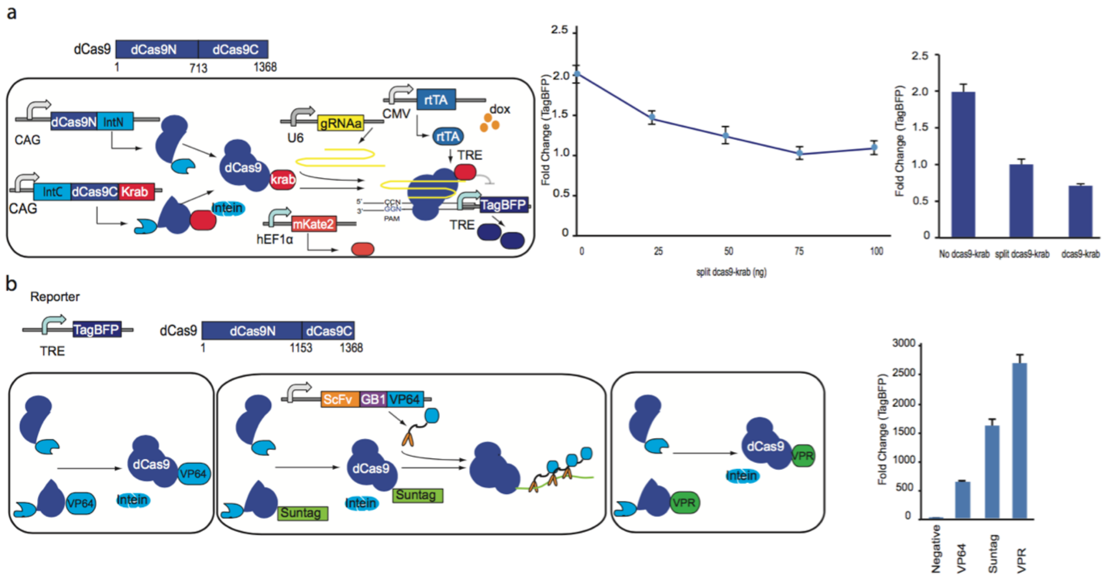


**Supplementary Figure 2 | Integration of split dCas9 constituents for transcriptional control**

**a.** Diagram of reconstitution of split dCas9 domains for transcription repression. The dCas9 constituents are split at residue 713. Repression efficiency was tested in HEK293 cells in response to varying amounts of plasmid DNA expressing split-dCas9 constituents. Each bar shows mean fold changes (mean ± SEM; n = 3) of TagBFP fluorescence measured by using flow cytometer 48 h after transfection. **b.** Different activation domains (VP64, Suntag and VPR) were fused to dCas9C. The dCas9 constituents are split at residue 1153. TagBFP was used as the reporter gene. Each bar shows mean fold changes (mean ± SEM; n = 3) of TagBFP fluorescence measured by using flow cytometer 48 h after transfection in HEK293 cells.

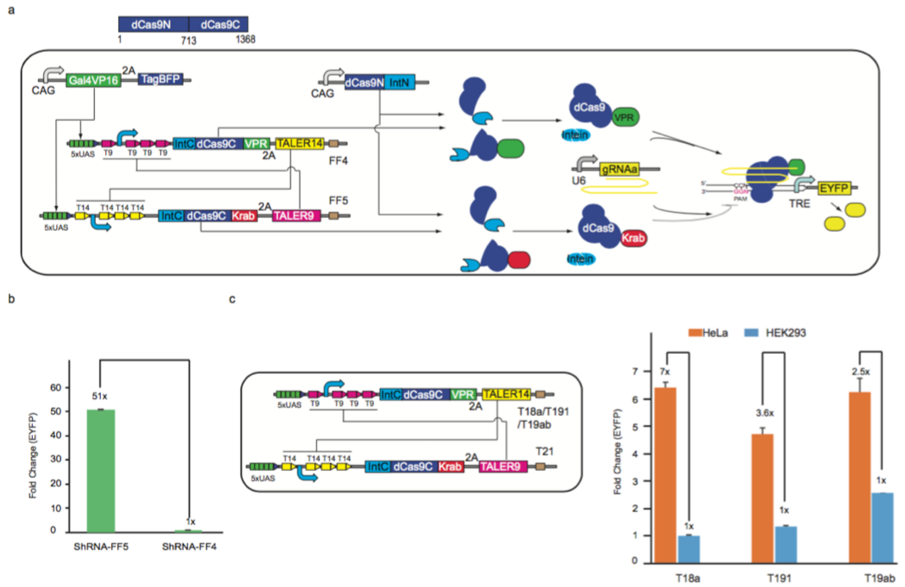


**Supplementary Figure 3 | Two-input and one-output sensory switch by swapping split dCas9 domains**

**a.** Schematic representation of a sensory switches for exchange of split-dCas9 activation and repression domains. The dCas9 constituents are split at residue 713. **b.** Control of the states of the sensory switch by shRNA-FF4 or shRNA-FF5. **c.** Setting states of sensory switches by endogenous microRNAs. For simplicity, only the core of the sensory switch is shown in the left panel. Performance of sensory switch in response to different microRNAs is shown in the right panel. **b. and c.** Data shown as the mean fold change ± SEM; n = 3) of EYFP fluorescence, was measured 48 h after transfection .

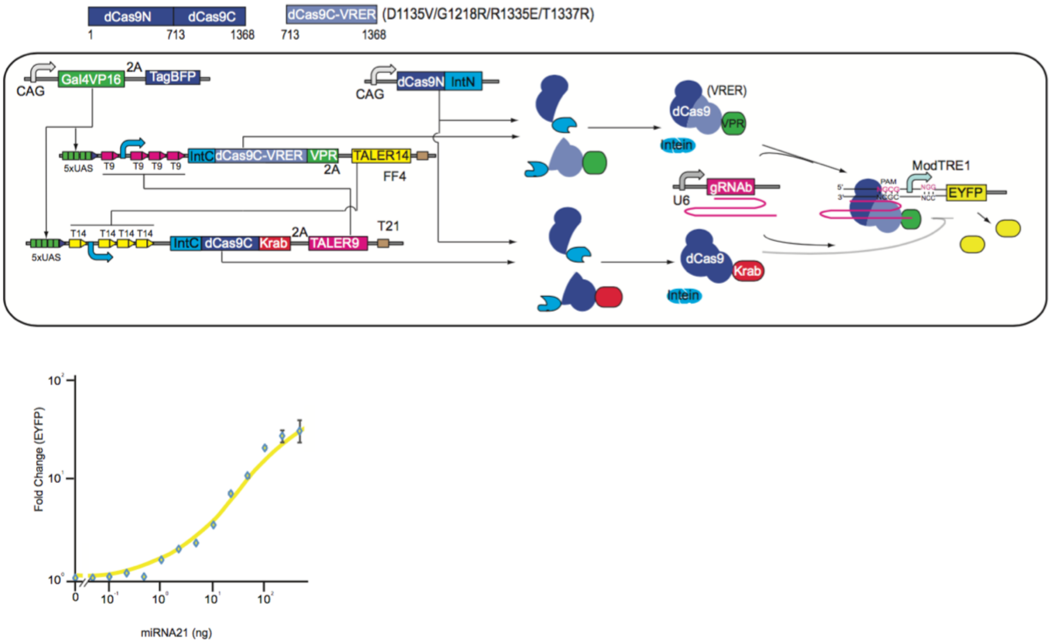


**Supplementary Figure 4 | Response of the sensory switch to miR21.**

Schematic representation of a sensory switches controlled by shRNA-FF4 and miR21is shown in the upper panel, and the response of sensory switch to varying amount of miR21 inputs is shown in the lower panel. The dCas9 constituents are split at residue 713. The light blue rectangle represents the mutant dCas9 domain (D1135V/G1218R/R1335E/T1337R, or VRER in short) that can recognize the NGCG PAM sequences but not the NGG PAM sequences. The ModTRE1 promoter contains 7 repeats of gRNAb binding sites with the NGCG PAM sequences upstream of a miniCMV core, followed by a triplicate of gRNAb binding sites with the NGG PAM sequences. The solid line was plotted by using qplot function in R package. Data shown as the mean fold change (mean ± SEM; n = 3) of EYFP fluorescence, was measured 48 h after transfection.

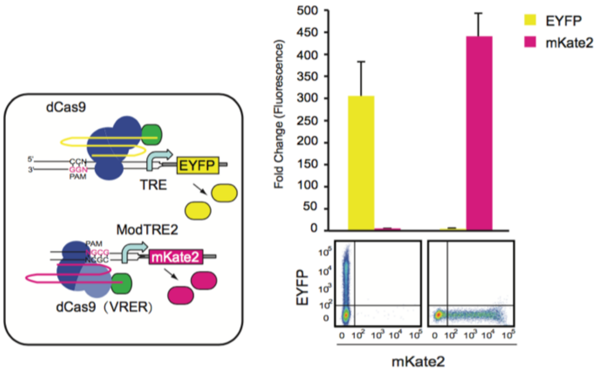


**Supplementary Figure 5 | Orthogonality test of the wild-type and mutant dCas9.** Schematic representation of orthogonality test is shown in the left panel. The dCas9 can recognize the NGG PAM sequence, and then activate EYFP. The mutant dCas9 that contains 4 point mutations (D1135V/G1218R/R1335E/T1337R, or VRER in short) can recognize the NGCG PAM sequence, and then activate mKate2. The right panel shows the fluorescence intensity of EYFP and mKate2 in a co-transfection experiment. Each bar shows mean fold change (mean ± SEM; n = 3) of EYFP or mKate2 fluorescence measured by using flow cytometer 48 h after transfection in HEK293 cells. The lower panel shows representative flow cytometry scatter plots.

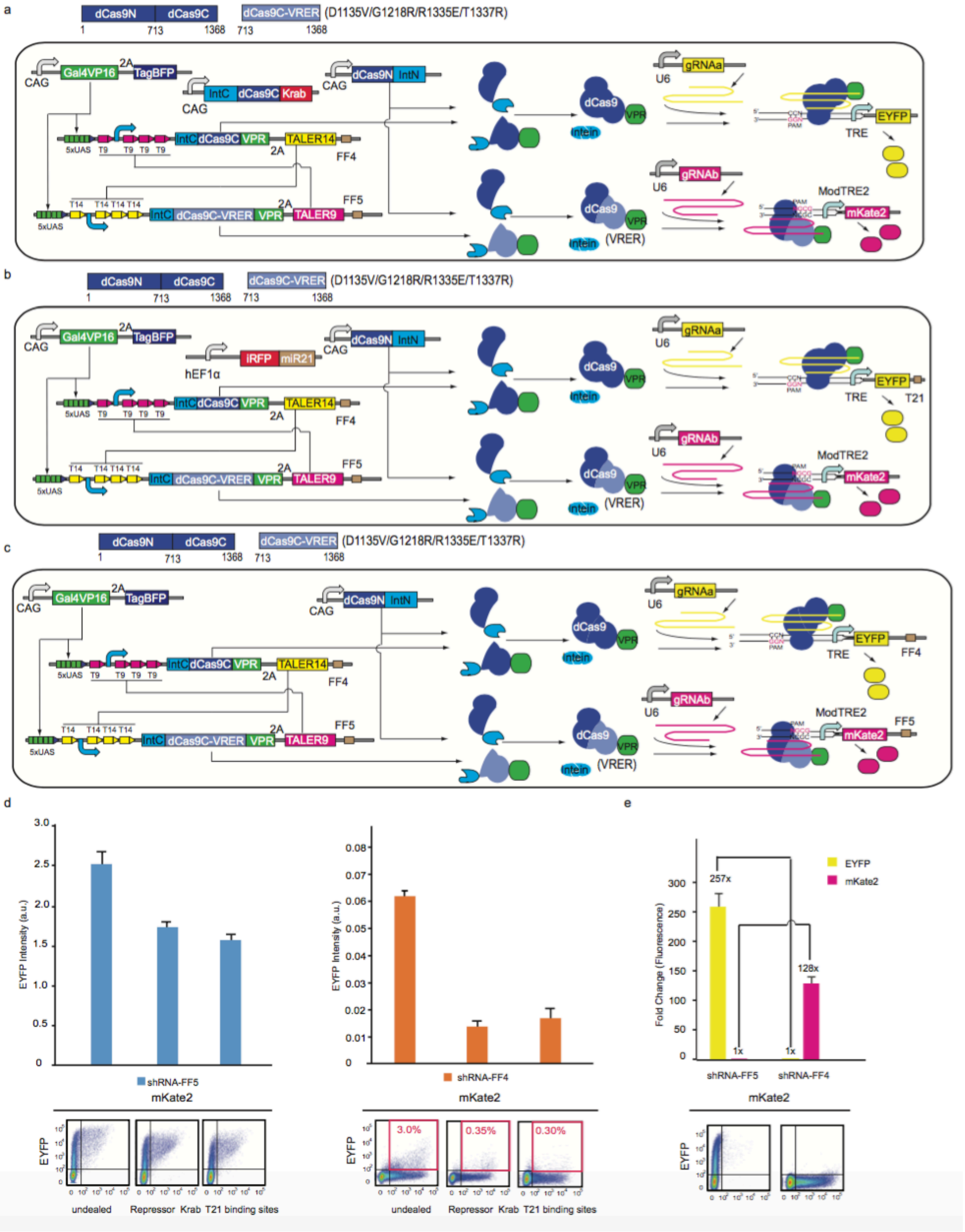

**Supplementary Figure 6 | Solution to leakiness problem in Figure 4.**

**a. b. and c.** Schematic representation of sensory switches for exchanging different dCas9 activation domains. Three different methods are shown to reduce the leaky expression of EYFP in the OFF state. **a.** A trace of dCas9:Krab was reconstituted to exert a weak transcriptional repression on the EYFP expression**. b.** Four tandem repeats of miR21 target sites were fused to the 3'-UTR of the EYFP reporter gene. Adding a trace of miR21 to apply a weak post-transcriptional repression on the EYFP expression**. c.** The EYFP reporter gene was fused with four tandem repeats of FF4 target sites in the 3'-UTR and the mKate2 reporter gene was fused with four tandem repeats of FF5

target sites in the 3’-UTR. **d.** Experimental results of panel **a.** and **b.** Each bar shows mean fold changes (mean ± SEM; n = 3) of EYFP fluorescence measured by using flow cytometer 48 h after transfection in HEK293 cells. The lower panel shows representative flow cytometry scatter plots. The percentage of EYFP-leaky expressed cells is shown in the red rectangle in the scatter plots. **e.** Experimental results of panel **c.** Each bar shows mean fold change (mean ± SEM; n = 3) of EYFP or mKate2 fluorescence measured by using flow cytometer 48 h after transfection in HEK293 cells. The lower panel shows representative flow cytometry scatter plots.

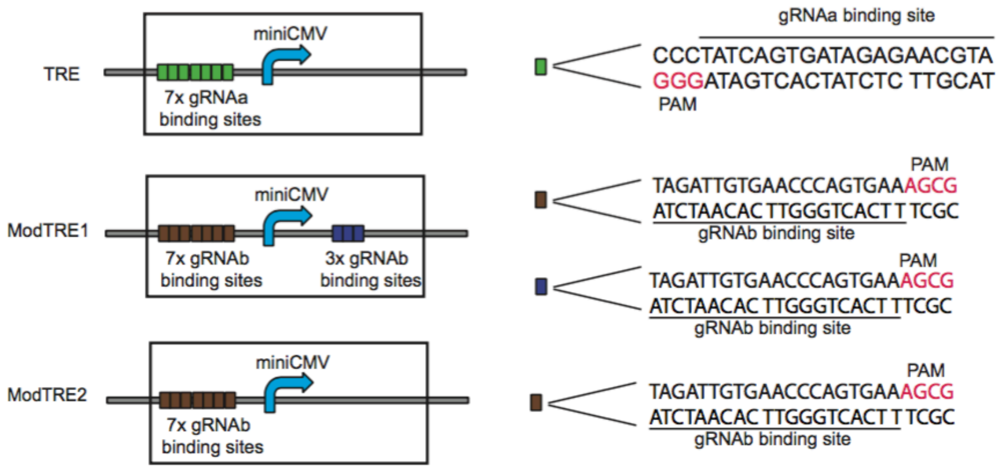


**Supplementary Figure 7 | Diagram of TRE promoter and derivatives**.

Schematic representation of the TRE and derivative promoters used in this study. The TRE promoter contains the NGG PAM and 7 repeats of gRNAa binding sites. The ModTRE1 contains the NGCG PAM and 7 repeats of gRNAb binding sites upstream of the minCMV core sequence, followed by the NGG PAM and triplicate of gRNAb binding sites. The ModTRE2 promoter has the NGCG PAM and 7 repeats of gRNAb binding sites upstream of miniCMV.

**Supplementary Table 1. The gRNA sequence used in this study.**

| Name | sequence |
|---|---|
| gRNAa | TACGTTCTCTATCACTGATA |
| gRNAb | TAGATTGTGAACCCAGTGAA |

**Supplementary Table 2. Primer sequence used in this study.**

| | |
|---|---|
| p1 | CGGCTCACCCGGGGGTGGCGGCTCACGAGACCGGATCCGCGCAACGCAATTAATGTGAG |
| p2 | CCACCACTAGTTGAGACCGTCGACTTACCATTCGCCA |
| p3 | AGTCGAGTGGTCTCATAGTGTCGCCTCCCAGTTGGGACAGGTCAA |
| p4 | AGCGATTCTAGACGGAAAGTGGACGGCATTGG |
| p5 | TCTAGTCTCGAGCAGGCTGAAGTTAGTAGCTCCGC |
| pScFVF | ATCAGAGGTCTCACTCAATGGGCCCCGACATCGTGATGA |
| pScFVR | AGTCAGGGTCTCATGGGACAACTCCAGTGAAAAGTTCTTCTC |
| pLacZR | CCTCCAATTGACTAGTACTAGTTGAGACCGTCGACTTACCATTCGCCA |
| pVP64F | ATCAGTAGGTCTCAGCGGCTCAGCTGACCCCAAGAAGAAGAG |
| pVP64R | TTAAGGCCGGTCTCATAGTTATTGTCTCCTTCCGTGTTTCAGT |
| pKrabR | TAGCGATGGTCTCATAGTTGACTCGAGCACAGATGACTTGATCTCGAAAG |
| p12 | AGTAGGGTCTCAGTCTCCCCTACCGTCGCCTATTCCGT |
| p13 | GGAGTCGGTCTCAAGACAAATCCTCCGTATTTCTTAGGGT |
| p14 | AGTAGGGTCTCACTCTGGCGCTAGCGAGCATTCTCTTCCT |
| p15 | GAGTCGGTCTCAAGAGAGCTCCAGAAAGGAAATGAGCTGG |
| p16 | AGTAGCGGGTCTCATGGACCTGTACTCCTTCCTATCAATGGTTGTATCGAAGT |
| p17 | AGTAGCGGTCTCATCCACCAAGGAGGTGCTGGATGCTAC |
| p889 | TTATCGTGGTCTCATAGTTCAGTTATCTAGTCTCGAGT |
| p888 | ATCACATGGTCTCACTCAAGTGGCCAAGGCGATTCCCTCCAT |
| p887 | ATCACATGGTCTCACAGCAGTGGCCAAGGCGATTCCCTCCAT |
| p886 | ATGGACTAGGTCTCAGTGATGAGCTTAGCGTTGAGGA |
| p885 | ATGATCGGTCTCATCACCCAAAGAAAGTTCGACA |
| p884 | TTATCGTGGTCTCATAGTGACCTGAGCCTTCTGAATATCTT |
| p883 | TTATCGTGGTCTCAGGCAGACCTGAGCCTTCTGAATATCTT |
| p882 | ATCACATGGTCTCACTCATCCGGCGTGGATGCCAAGGCCAT |
| p881 | ATCACATGGTCTCACTCAAGCGAGGAAACAATCACACC |
| p880 | ATCACATGGTCTCACTCAAGTAAAAAGCTCAAGAGCGTCAAGGAGC |
| p879 | ATCACATGGTCTCACAGCAGCGAGGAAACAATCACACC |
| p878 | TTATCGTGGTCTCATAGTCTTCCTTGTCATCCAGGCGAA |
| p877 | TTATCGTGGTCTCAGGCACTTCCTTGTCATCCAGGCGAA |
| p876 | ATCACATGGTCTCACAGCAGTAAAAAGCTCAAGAGCGTCAAGGAGC |
| p875 | TTATCGTGGTCTCATAGTCTTGCCCTTCTCCACCTTAGCG |
| p874 | TTATCGTGGTCTCAGGCACTTGCCCTTCTCCACCTTAGCG |
| p873 | ATCACATGGTCTCACAGCAGTGGCGTGGATGCCAAGGCCAT |

| | |
|---|---|
| p872 | TTATCGTGGTCTCATAGTAGCATTGATGGGGTTCTCCTCG |
| p871 | TTATCGTGGTCTCAGGCAAGCATTGATGGGGTTCTCCTCG |
| p870 | ATCACATGGTCTCACTCAATGGACAAGAAGTACTCCATTGGCCT |
| p812r | ATACCAGGTCTCATAGTAAACAAACTTGTGTCAAATATACTAAGCCCGGTG |
| gRNAa1 | ACCGTACGTTCTCTATCACTGATA |
| gRNAa2 | AAACTATCAGTGATAGAGAACGTA |
| gRNAb1 | ACCGTAGATTGTGAACCCAGTGAA |
| gRNAb2 | AAACTTCACTGGGTTCACAATCTA |
| p18 | AGTAGCGGGTCTCATAGTTGTTATGTGTCTGGGAGGTATTGGCTTCTAG |
| p701 | AGTAGCGGGTCTCAGACTCAAATCCTCCGTATTTCTTAGGGTCCCAGTCCTTCTTC |
| p702 | AGTCGGGTCTCAAGTCCCCTACCGTCGCCTATTCCGTCCTC |
| p703 | AGTCGAGGTCTCACTGCTTCCTATCAATGGTTGTATCGAAGT |
| p704 | AGTCGGGTCTCAGCAGTACAGGTCCACCAAGGAGGTGCTGGATGCTAC |
| M13F | GCCAGGGTTTTCCCAGTCACGA |

## Supplementary Table 3. Transfection configuration.

| Plasmid DNA used in Figure 1a | Intein-split dCas9 + gRNA | Directly-split dCas9 + gRNA | Fμll length dCas9 + gRNA | Negative |
|---|---|---|---|---|
| pCAG-dCas9N:IntN | 100 ng | | | |
| pCAG-IntC:dCas9C:VPR | 100 ng | | | |
| pCAG-dCas9N | | 100 ng | | |
| pCAG-dCas9C:VPR | | 100 ng | | |
| pCAG-dCas9:VPR | | | 100 ng | |
| pSIREN_U6-gRNAa | 100 ng | 100 ng | 100 ng | 100 ng |
| phEF1α-mKate2 | 100 ng | 100 ng | 100 ng | 100 ng |
| pTRE-TagBFP | 100 ng | 100 ng | 100 ng | 100 ng |
| pDT7004 | | | 100 ng | 200 ng |
| Four pairs of split-dCas9 plasmid DNAs were used with the same amount as shown above | | | | |

| Plasmid DNA used in Figure 1b | 1 | 2 | 3 | 4 |
|---|---|---|---|---|
| pCAG-dCas9N(1-203):IntN | 100 ng | 100 ng | | |
| pCAG-IntC:dCas9C(204-1368):VPR | 100 ng | | 100 ng | |
| pCAG-dCas9N(1-468):IntN | | | 100 ng | 100 ng |
| pCAG-IntC:dCas9C(469-1368):VPR | | 100 ng | | 100 ng |
| pSIREN_U6-gRNAa | 100 ng | 100 ng | 100 ng | 100 ng |

| | | | | |
|---|---|---|---|---|
| phEF1α-mKate2 | 100 ng | 100 ng | 100 ng | 100 ng |
| pTRE-TagBFP | 100 ng | 100 ng | 100 ng | 100 ng |
| Other pairs of split-dCas9 plasmid DNAs were used with the same amount as shown above | | | | |

| Plasmid DNA used in Figure 1c | 1 | 2 | 3 | 4 |
|---|---|---|---|---|
| pCAG-dCas9N(1-713):IntN | 100 ng | | | 100 ng |
| pCAG-IntC:dCas9C(714-1368):VPR | 100 ng | | 100 ng | |
| pCAG-dCas9N(1-1153)(D1153E):IntN | | 100 ng | 100 ng | |
| pCAG-dCas9C(1154-1368)(R1335Q/T1337R):VPR | | 100 ng | | 100 ng |
| pSIREN_U6-gRNAa | 100 ng | 100 ng | 100 ng | 100 ng |
| pSIREN_U6-gRNAb | 100 ng | 100 ng | 100 ng | 100 ng |
| pModTRE2-mKate2 | 100 ng | 100 ng | 100 ng | 100 ng |
| pTRE-EYFP | 100 ng | 100 ng | 100 ng | 100 ng |
| pCAG-TagBFP | 100 ng | 100 ng | 100 ng | 100 ng |

| Plasmid DNA used in Figure 2a | 1 | 2 | 3 | 4 | 5 | 6 | 7 | 8 |
|---|---|---|---|---|---|---|---|---|
| pCAG-dCas9N(1-1153):IntN | 100 ng | 100 ng | 100 ng | | 100 ng | | | |
| pCAG-IntC:dCas9C(1154-1368):Suntag | 100 ng | 100 ng | | 100 ng | | 100 ng | | |
| Rapalog | 0.5 μM | | 0.5 μM | 0.5 μM | | | 0.5 μM | |
| pCAG-Scfv:GB1:FKBP | 100 ng | 100 ng | 100 ng | 100 ng | 100 ng | 100 ng | 100 ng | 100 ng |
| pCAG-FRB*:VP64 | 100 ng | 100 ng | 100 ng | 100 ng | 100 ng | 100 ng | 100 ng | 100 ng |
| pSIREN_U6-gRNAa | 100 ng | 100 ng | 100 ng | 100 ng | 100 ng | 100 ng | 100 ng | 100 ng |
| phEF1α-mKate2 | 100 ng | 100 ng | 100 ng | 100 ng | 100 ng | 100 ng | 100 ng | 100 ng |
| pTRE-TagBFP | 100 ng | 100 ng | 100 ng | 100 ng | 100 ng | 100 ng | 100 ng | 100 ng |
| pDT7004 | | | 100 ng | 100 ng | 100 ng | 100 ng | 200 ng | 200 ng |

| Plasmid DNA used in Figure 2b | 1 | 2 | 3 | 4 | 5 | 6 | 7 | 8 |
|---|---|---|---|---|---|---|---|---|
| pCAG-dCas9N(1-713) | 100 ng | 100 ng | 100 ng | | 100 ng | | | |
| pCAG-dCas9M(714-1153):IntN | 100 ng | 100 ng | | 100 ng | | 100 ng | | |
| pCAG-IntC:dCas9C(1154-1368):Suntag | 100 ng | | 100 ng | 100 ng | | | 100 ng | |
| pCAG-ScFv:VP64 | 100 ng | 100 ng | 100 ng | 100 ng | 100 ng | 100 ng | 100 ng | 100 ng |
| pSIREN_U6-gRNAa | 100 ng | 100 ng | 100 ng | 100 ng | 100 ng | 100 ng | 100 ng | 100 ng |
| phEF1α-mKate2 | 100 ng | 100 ng | 100 ng | 100 ng | 100 ng | 100 ng | 100 ng | 100 ng |
| pTRE-TagBFP | 100 ng | 100 ng | 100 ng | 100 ng | 100 ng | 100 ng | 100 ng | 100 ng |
| pDT7004 | | 100 ng | 100 ng | 100 ng | 200 ng | 200 ng | 200 ng | 300 ng |

| Plasmid DNA used in Figure 3b | 1-1 | 2-1 | 3-1 | 4-1 | 1-2 | 2-2 | 3-2 | 4-2 |
|---|---|---|---|---|---|---|---|---|
| pCAG-Gal4VP16-2A-TagBFP | 100 ng | 100 ng | 100 ng | 100 ng | 100 ng | 100 ng | 100 ng | 100 ng |
| pUASx5-T9+T9x3-IntC:dCas9C(714-1368)VRER:VPR-2A-TALER14-4xTarget^FF4 | 100 ng | 100 ng | | 100 ng | 100 ng | 100 ng | | 100 ng |
| pUASx5-T14+T14x3-IntC:dCas9C(714-1368):Krab-2A-TALER9-4xTarget^FF5 | 100 ng | | 100 ng | 100 ng | 100 ng | | 100 ng | 100 ng |
| pCAG-dCas9N(1-713):IntN | 100 ng | 100 ng | 100 ng | | 100 ng | 100 ng | 100 ng | |
| pSIREN_U6-gRNAb | 100 ng | 100 ng | 100 ng | 100 ng | 100 ng | 100 ng | 100 ng | 100 ng |
| pModTRE1-EYFP | 100 ng | 100 ng | 100 ng | 100 ng | 100 ng | 100 ng | 100 ng | 100 ng |
| pSIREN_U6-shRNA-FF5 | 100 ng | 100 ng | 100 ng | 100 ng | | | | |
| pSIREN_U6-shRNA-FF4 | | | | | 100 ng | 100 ng | 100 ng | 100 ng |

| | | | | | | | | |
|---|---|---|---|---|---|---|---|---|
| pDT7004 | | 100 ng | 100 ng | 100 ng | | 100 ng | 100 ng | 100 ng |

| Plasmid DNA used in Figure 3c | Amount |
|---|---|
| pCAG-Gal4VP16-2A-TagBFP | 100 ng |
| pUASx5-T9+T9x3-IntC:dCas9C(714-1368)VRER:VPR-2A-TALER14-4xTarget^FF4 | 100 ng |
| pUASx5-T14+T14x3-IntC:dCas9C(714-1368):Krab-2A-TALER9-4xTarget^FF5 | 100 ng |
| pCAG-dCas9N(1-713):IntN | 100 ng |
| pSIREN_U6-gRNAb | 100 ng |
| pModTRE1-EYFP | 100 ng |
| pSIREN_U6-shRNA-FF5 | z ng |
| pDT7004 | 200-z ng |
| z=0, 0.05, 0.1, 0.2, 0.5, 1, 2, 5, 10, 20, 50, 100, 200 | |

| Plasmid DNA used in Figure 3d | Amount |
|---|---|
| pCAG-Gal4VP16-2A-TagBFP | 100 ng |
| pUASx5-T9+T9x3-IntC:dCas9C(714-1368)VRER:VPR-2A-TALER14-4xTarget^miR18a | 100 ng |
| pUASx5-T14+T14x3-IntC:dCas9C(714-1368):Krab-2A-TALER9-4xTarget^miR21 | 100 ng |
| pCAG-dCas9N(1-713):IntN | 100 ng |
| pSIREN_U6-gRNAb | 100 ng |
| pModTRE1-EYFP | 100 ng |

| Plasmid DNA used in Figure 4b | 1 | 2 | 3 | 4 |
|---|---|---|---|---|
| pCAG-Gal4VP16-2A-TagBFP | 100 ng | 100 ng | 100 ng | 100 ng |
| pUASx5-T9+T9x3-IntC:dCas9C(714-1368):VPR-2A-TALER14-4xTarget^FF4 | 100 ng | 100 ng | 100 ng | 100 ng |
| pUASx5-T14+T14x3-IntC:dCas9C(714-1368)VRER:VPR-2A-TALER9-4xTarget^FF5 | 50 ng | 50 ng | 50 ng | 50 ng |
| pCAG-dCas9N(1-713):IntN | 100 ng | 100 ng | 100 ng | |
| pSIREN_U6-gRNAa | 80 ng | 80 ng | 80 ng | 80 ng |
| pSIREN_U6-gRNAb | 80 ng | 80 ng | 80 ng | 80 ng |
| pModTRE2-mKate2 | 100 ng | 100 ng | 100 ng | 100 ng |
| pTRE-EYFP | 100 ng | 100 ng | 100 ng | 100 ng |
| pSIREN_U6-shRNA-FF5 | 100 ng | | | |
| pSIREN_U6-shRNA-FF4 | | 100 ng | | |
| pDT7004 | | | 100 ng | 200 ng |

| Plasmid DNA used in Figure 4c | 1 |
|---|---|
| pCAG-Gal4VP16-2A-TagBFP | 100 ng |
| pUASx5-T9+T9x3-IntC:dCas9C(714-1368):VPR-2A-TALER14-4xTarget^FF4 | 100 ng |
| pUASx5-T14+T14x3-IntC:dCas9C(714-1368)VRER:VPR-2A-TALER9-4xTarget^FF5 | 50 ng |
| pCAG-dCas9N(1-713):IntN | 100 ng |
| pSIREN_U6-gRNAa | 80 ng |
| pSIREN_U6-gRNAb | 80 ng |
| pModTRE2-mKate2 | 100 ng |
| pTRE-EYFP | 100 ng |
| pSIREN_U6-shRNA-FF4 | z ng |
| pDT7004 | 200-z ng |
| z=0, 0.05, 0.1, 0.2, 0.5, 1, 2, 5, 10, 20, 50, 100, 200 | |

| Plasmid DNA used in Supplementary Figure. 1c | Intein-split Cas9N + Cas9C + gRNA | Directly-split Cas9N + Cas9C + gRNA | Intein-split Cas9N + gRNA | Intein-split Cas9C + gRNA | Intein-split Cas9N +Cas9C | Full length Cas9 + gRNA | Full length Cas9 |
|---|---|---|---|---|---|---|---|
| pCAG-Cas9N:IntN | 100 ng | | 100 ng | | 100 ng | | |
| pCAG-IntC:Cas9C | 100 ng | | | 100 ng | 100 ng | | |
| pCAG-Cas9N | | 100 ng | | | | | |
| pCAG-Cas9C | | 100 ng | | | | | |
| pCAG-Cas9 | | | | | | 100 ng | 100 ng |
| pSIREN_U6-gRNAb | 100 ng | 100 ng | 100 ng | 100 ng | | 100 ng | |
| phEF1α-EYFP | 100 ng | 100 ng | 100 ng | 100 ng | 100 ng | 100 ng | 100 ng |
| pCAG-TagBFP | 100 ng | 100 ng | 100 ng | 100 ng | 100 ng | 100 ng | 100 ng |
| pDT7004 | | | 100 ng | 100 ng | 100 ng | 100 ng | 200 ng |
| Four pairs of split-Cas9 plasmid DNAs were used with the same amount as shown above | | | | | | | |

| Plasmid DNA used in Supplementary Figure 2a (middle panel) | Amount |
|---|---|
| pCAG-dCas9N(1-713):IntN | z ng |
| pCAG-IntC:dCas9C(714-1368):Krab | z ng |
| pSIREN_U6-gRNAa | 100 ng |
| phEF1α-mKate2 | 100 ng |
| pTRE-TagBFP | 100 ng |
| pDT7004 | 200-2z ng |
| pCMV-rtTA | 100 ng |
| Dox | 50 ng/mL |
| z = 0, 25, 50, 75, 100 | |

| Plasmid DNA used in Supplementary Figure 2a (right panel) | 1 | 2 | 3 |
|---|---|---|---|
| pCAG-dCas9N(1-713):IntN | | 100 ng | |
| pCAG-IntC:dCas9C(714-1368):Krab | | 100 ng | |
| pCAG-dCas9:Krab | | | 100 ng |

| | | | |
|---|---|---|---|
| pSIREN_U6-gRNAa | 100 ng | 100 ng | 100 ng |
| phEF1α-mKate2 | 100 ng | 100 ng | 100 ng |
| pTRE-TagBFP | 100 ng | 100 ng | 100 ng |
| pDT7004 | 200 ng | | 100 ng |
| pCMV-rtTA | 100 ng | 100 ng | 100 ng |
| Dox | 50 ng/mL | 50 ng/mL | 50 ng/mL |

| Plasmid DNA used in Supplementary Figure 2b | 1 | 2 | 3 | 4 |
|---|---|---|---|---|
| pCAG-dCas9N(1-1153):IntN | | 100 ng | 100 ng | 100 ng |
| pCAG-IntC:dCas9C(1154-1368):VP64 | | 100 ng | | |
| pCAG-IntC:dCas9C(1154-1368):Suntag | | | 100 ng | |
| pCAG-IntC:dCas9C(1154-1368):VPR | | | | 100 ng |
| pCAG-ScFv:GB1:VP64 | | | 100 ng | |
| pSIREN_U6-gRNAa | 100 ng | 100 ng | 100 ng | 100 ng |
| phEF1α-mKate2 | 100 ng | 100 ng | 100 ng | 100 ng |
| pTRE-TagBFP | 100 ng | 100 ng | 100 ng | 100 ng |
| pDT7004 | 300 ng | 100 ng | | 100 ng |

| Plasmid DNA used in Supplementary Figure 3b | 1 | 2 |
|---|---|---|
| pCAG-Gal4VP16-2A-TagBFP | 100 ng | 100 ng |
| pUASx5-T9+T9x3-IntC:dCas9C(714-1368):VPR-2A-TALER14-4xTarget^FF4 | 100 ng | 100 ng |
| pUASx5-T14+T14x3-IntC:dCas9C(714-1368):Krab-2A-TALER9-4xTarget^FF5 | 100 ng | 100 ng |
| pCAG-dCas9N(1-713):IntN | 100 ng | 100 ng |
| pSIREN_U6-gRNAa | 100 ng | 100 ng |
| pTRE-EYFP | 100 ng | 100 ng |
| pSIREN_U6-shRNA-FF5 | 100 ng | |

| | | |
|---|---|---|
| pSIREN_U6-shRNA-FF4 | | 100 ng |

| Plasmid DNA used in Supplementary Figure 3c | 1 | 2 | 3 |
|---|---|---|---|
| pCAG-Gal4VP16-2A-TagBFP | 100 ng | 100 ng | 100 ng |
| pUASx5-T9+T9x3-IntC:dCas9C(714-1368):VPR-2A-TALER14-4xTarget^miR18a | 100 ng | | |
| pUASx5-T9+T9x3-IntC:dCas9C(714-1368):VPR-2A-TALER14-4xTarget^miR191 | | 100 ng | |
| pUASx5-T9+T9x3-IntC:dCas9C(714-1368):VPR-2A-TALER14-4xTarget^miR19ab | | | 100 ng |
| pUASx5-T14+T14x3-IntC:dCas9C(714-1368):Krab-2A-TALER9-4xTarget^miR21 | 100 ng | 100 ng | 100 ng |
| pCAG-dCas9N(1-713):IntN | 100 ng | 100 ng | 100 ng |
| pSIREN_U6-gRNAa | 100 ng | 100 ng | 100 ng |
| pTRE-EYFP | 100 ng | 100 ng | 100 ng |

| Plasmid DNA used in Supplementary Figure 4 | 1 |
|---|---|
| pCAG-Gal4VP16-2A-TagBFP | 100 ng |
| pUASx5-T9+T9x3-IntC:dCas9C(714-1368)VRER:VPR-2A-TALER14-4xTarget^FF4 | 100 ng |
| pUASx5-T14+T14x3-IntC:dCas9C(714-1368):Krab-2A-TALER9-4xTarget^miR21 | 100 ng |
| pCAG-dCas9N(1-713):IntN | 100 ng |
| pSIREN_U6-gRNAb | 100 ng |

| | |
|---|---|
| pModTRE1-EYFP | 100 ng |
| phEF1α-iRFP-miRNA21 | z ng |
| pDT7004 | 400-z ng |
| z=0, 0.05, 0.1, 0.2, 0.5, 1, 2, 5, 10, 20, 50, 100, 200, 400 | |

| Plasmid DNA used in Supplementary Figure 5 | 1 | 2 |
|---|---|---|
| pCAG-Gal4VP16-2A-TagBFP | 100 ng | 100 ng |
| pUASx5-T9+T9x3-IntC:dCas9C(714-1368):VPR-2A-TALER14-4xTarget^FF4 | 100 ng | |
| pUASx5-T14+T14x3-IntC:dCas9C(714-1368)VRER:VPR-2A-TALER9-4xTarget^FF5 | | 100 ng |
| pCAG-dCas9N(1-713):IntN | 100 ng | 100 ng |
| pSIREN_U6-gRNAa | 100 ng | 100 ng |
| pSIREN_U6-gRNAb | 100 ng | 100 ng |
| pModTRE2-mKate2 | 100 ng | 100 ng |
| pTRE-EYFP | 100 ng | 100 ng |

| Plasmid DNA used in Supplementary Figure 6d | 1 | 2 | 3 | 4 | 5 | 6 |
|---|---|---|---|---|---|---|
| pCAG-Gal4VP16-2A-TagBFP | 100 ng | 100 ng | 100 ng | 100 ng | 100 ng | 100 ng |
| pUASx5-T9+T9x3-IntC:dCas9C(714-1368):VPR-2A-TALER14-4xTarget^FF4 | 100 ng | 100 ng | 100 ng | 100 ng | 100 ng | 100 ng |
| pUASx5-T14+T14x3-IntC:dCas9C(714-1368)VRER:VPR-2A-TALER9-4xTarget^FF5 | 50 ng | 50 ng | 50 ng | 50 ng | 50 ng | 50 ng |
| pCAG-dCas9N(1-713):IntN | 100 ng | 100 ng | 100 ng | 100 ng | 100 ng | 100 ng |
| pSIREN_U6-gRNAa | 80 ng | 80 ng | 80 ng | 80 ng | 80 ng | 80 ng |
| pSIREN_U6-gRNAb | 80 ng | 80 ng | 80 ng | 80 ng | 80 ng | 80 ng |
| pModTRE2-mKate2 | 100 ng | 100 ng | 100 ng | 100 ng | 100 ng | 100 ng |

| | | | | | | |
|---|---|---|---|---|---|---|
| pTRE-EYFP | 100 ng | 100 ng | 100 ng | 100 ng | | |
| pSIREN_U6-shRNA-FF5 | 100 ng | | 100 ng | | 100 ng | |
| pSIREN_U6-shRNA-FF4 | | 100 ng | | 100 ng | | 100 ng |
| pCAG-IntC:dCas9C(714-1368):Krab | | | 100 ng | 100 ng | | |
| pTRE-EYFP-4*T21 | | | | | 100 ng | 100 ng |
| phEF1α-iRFP-miR21 | | | | | 100 ng | 100 ng |
| pDT7004 | 100 ng | 100 ng | | | | |

| Plasmid DNA used in Supplementary Figure 6e | 1 | 2 |
|---|---|---|
| pCAG-Gal4VP16-2A-TagBFP | 100 ng | 100 ng |
| pUASx5-T9+T9x3-IntC:dCas9C(714-1368):VPR-2A-TALER14-4xTarget^FF4 | 100 ng | 100 ng |
| pUASx5-T14+T14x3-IntC:dCas9C(714-1368)VRER:VPR-2A-TALER9-4xTarget^FF5 | 50 ng | 50 ng |
| pCAG-dCas9N(1-713):IntN | 100 ng | 100 ng |
| pSIREN_U6-gRNAa | 80 ng | 80 ng |
| pSIREN_U6-gRNAb | 80 ng | 80 ng |
| pModTRE2-mKate2-4xTarget^FF5 | 100 ng | 100 ng |
| pTRE-EYFP-4xTarget^FF4 | 100 ng | 100 ng |
| pSIREN_U6-shRNA-FF5 | 100 ng | |
| pSIREN_U6-shRNA-FF4 | | 100 ng |

## Supplementary Methods

## Plasmid DNA Constructs

pDT7004, pCh055 (pCAG-Gal4VP16-2A-TagBFP), pCh023 (pSIREN_U6-shRNA-FF5), pCh022 (pSIREN_U6-shRNA-FF4) were described in our previous study[1]. pZ555 (phEF1α-mKate2) was described in our previous study[2]. pB116 (pTRE-TagBFP), pB115 (pCAG-TagBFP), pB044 (TRE-EYFP), pB016 (pCMV-rtTA), pB328 (phEF1α-iRFP-miRNA21), pB400 (pTRE-EYFP-4*T21), pYB050 (phEF1α-EYFP) were obtained from the Syngentech company.

pDS42 (pSIREN_U6-gRNAa) was made by inserting gRNAa into pY227 (pSIREN_U6-BsaI-Lacz-BsaI-gRNA backbone) in a Golden Gate reaction with BsaI.. pY227 was obtained from the Syngentech company.

pY239 (pSIREN_U6-gRNAb) was made by inserting gRNAb into pY227 (pSIREN_U6-BsaI-Lacz-BsaI-gRNA backbone) in a Golden Gate reaction with BsaI.

pDS001 (pEntr_NLS_BsaI-LacZ-BsaI_NLS) was made by inserting BsaI-LacZ-BsaI fragment into pT096 (pENTR_NLS_dCas9) with XmaI and SpeI. The BsaI-LacZ-BsaI fragment was PCR amplified from pUC19 vector using primers p1 and p2.

pA017 (CAG-NLS_BsaI-LacZ-BsaI_NLS) was prepared by combining pZ250 (pENTR_L4-CAG_L1)[2] and pDS001 with pT592[1] in a Gateway LR reaction.

pSA001 (CAG-NLS-Cas9(1-203)) was made by inserting Cas9(1-203) fragment into pA017 by Golden Gate assembly. The Cas9(1-203) fragment was PCR amplified from pT106 (CAG_Cas9) vector using primer p870 and p872.

pSA002 (CAG-NLS-Cas9(1-468)) was made by inserting Cas9(1-468) fragment into pA017 by Golden Gate assembly. The Cas9(1-468) fragment was PCR amplified from pT106 (CAG_Cas9) vector using primer p870 and p878.

pSA003 (CAG-NLS-Cas9(1-713)) was made by inserting Cas9(1-713) fragment into pA017 with BsaI. The Cas9(1-713) fragment was PCR amplified from pT106 (CAG_Cas9) vector using primer p870 and p884.

pSA004 (CAG-NLS-Cas9(1-1153)) was made by inserting Cas9(1-1153) fragment into pA017 with BsaI. The Cas9(1-203) fragment was PCR amplified from pT106 (CAG_Cas9) vector using primer p870 and p875.

pSA005 (BsaI-InteinN-BsaI) was synthetized in Genewiz by inserting inteinN fragment into pUC19 vector as the donor vector for InteinN fragment.

pSA006 (CAG-NLS-Cas9(1-203)-InteinN) was made in a Golden Gate reaction with Cas9(1-203) fragment, pSA005 (the donor vector for InteinN) and pA017. The Cas9(1-203) fragment was PCR amplified from pT106 (CAG_Cas9) vector using primer p870 and p871.

pSA007 (CAG-NLS-Cas9(1-468)-InteinN) was made in a Golden Gate reaction with Cas9(1-468) fragment, pSA005 (the donor vector for InteinN) and pA017. The Cas9(1-468) fragment was PCR amplified from pT106 (CAG_Cas9) vector using primer p870 and p877.

pSA008 (CAG-NLS-Cas9(1-713)-InteinN) was made in a Golden Gate reaction with Cas9(1-713) fragment, pSA005 (the donor vector for InteinN) and pA017. The Cas9(1-713) fragment was PCR amplified from pT106 (CAG_Cas9) vector using primer p870 and p883.

pSA009 (CAG-NLS-Cas9(1-1153)-InteinN) was made in a Golden Gate reaction with Cas9(1-1153) fragment, pSA005 (the donor vector for InteinN) and pA017. The Cas9(1-1153) fragment was PCR amplified from pT106(CAG_Cas9) vector using primer p870 and p874.

pSA010 (CAG-NLS-dCas9(1-203)) was made in a Golden Gate reaction with dCas9(1-203) fragment and pA017. The dCas9(1-203) fragment was PCR amplified from pT096 (pENTR_NLS_dCas9) vector using primer p870 and p872.

pSA012 (CAG-NLS-dCas9(1-468)) was made in a Golden Gate reaction with dCas9(1-468) fragment and pA017. The dCas9(1-468) fragment was PCR amplified from pT096 (pENTR_NLS_dCas9) vector using primer p870 and p878.

pSA013 (CAG-NLS-dCas9(1-713)) was made in a Golden Gate reaction with dCas9(1-713) fragment and pA017. The dCas9(1-713) fragment was PCR amplified from pT096 (pENTR_NLS_dCas9) vector using primer p870 and p884.

pSA014 (CAG-NLS-dCas9(1-1153)) was made in a Golden Gate reaction with dCas9(1-1153) fragment and pA017. The dCas9(1-1153) fragment was PCR amplified from pT096 (pENTR_NLS_dCas9) vector using primer p870 and p875.

pSA015 (BsaI-InteinC-BsaI) was synthetized in Genewiz by inserting inteinC fragment into pUC19 vector as the donor vector for inteinC fragment.

pSA016 (CAG-NLS-dCas9(1-203)-InteinN) was made in a Golden Gate reaction with dCas9(1-203) fragment, pSA005 (the donor vector for InteinN) and pA017. The dCas9(1-203) fragment was PCR amplified from pT096 (pENTR_NLS_dCas9) vector using primer p870 and p871.

pSA017 (CAG-NLS-dCas9(1-468)-InteinN) was made in a Golden Gate reaction with dCas9(1-468) fragment, pSA005 (the donor vector for InteinN) and pA017. The dCas9(1-468) fragment was PCR amplified from pT096 (pENTR_NLS_dCas9) vector using primer p870 and p877.

pSA018 (CAG-NLS-dCas9(1-713)-InteinN) was made in a Golden Gate reaction with dCas9(1-713) fragment, pSA005 (the donor vector for InteinN) and pA017. The dCas9(1-713) fragment was PCR amplified from pT096 (pENTR_NLS_dCas9) vector using primer p870 and p883.

pSA019 (CAG-NLS-dCas9(1-1153)-InteinN) was made in a Golden Gate reaction with dCas9(1-1153) fragment, pSA005 (the donor vector for InteinN) and pA017. The dCas9(1-1153) fragment was PCR amplified from pT096 (pENTR_NLS_dCas9) vector using primer p870 and p874.

pSA020 (CAG-NLS-Cas9(204-1368)) was made in a Golden Gate reaction with Cas9(204-1368) fragment and pA017. The Cas9(204-1368) fragment was PCR amplified from pT106 (CAG_Cas9) vector using primer p882 and p3.

pSA021 (CAG-NLS-Cas9(469-1368)) was made in a Golden Gate reaction with Cas9(469-1368) fragment and pA017. The Cas9(469-1368) fragment was PCR amplified from pT106 (CAG_Cas9) vector using primer p881 and p3.

pSA022 (CAG-NLS-Cas9(714-1368)) was made in a Golden Gate reaction with Cas9(714-1368) fragment and pA017. The Cas9(714-1368) fragment was PCR amplified from pT106 (CAG_Cas9) vector using primer p888 and p3.

pSA023 (CAG-NLS-Cas9(1154-1368)) was made in a Golden Gate reaction with Cas9(1154-1368) fragment and pA017. The Cas9(1154-1368) fragment was PCR amplified from pT106 (CAG_Cas9) vector using primer p880 and p3.

pSA024 (CAG-NLS-dCas9(204-1368)-VPR) was made in a Golden Gate reaction with dCas9(204-1368-VPR) fragment and pA017. The dCas9(204-1368-VPR) fragment was PCR amplified from pA046 (CAG_dCas9_VPR) vector using primer p882 and p812r.

pSA025 (CAG-NLS-dCas9(469-1368)-VPR) was made in a Golden Gate reaction with dCas9(469-1368-VPR) fragment and pA017. The dCas9(1-468) fragment was PCR amplified from pA046 (CAG_dCas9_VPR) vector using primer p881 and p812r.

pSA026 (CAG-NLS-dCas9(714-1368)-VPR) was made in a Golden Gate reaction with dCas9 (714-1368-VPR) fragment and pA017. The dCas9(714-1368-VPR) fragment was PCR amplified from pA046(CAG_dCas9_VPR) vector using primer

p888 and p812r.

pSA027 (CAG-NLS-dCas9(1154-1368)-VPR) was made in a Golden Gate reaction with dCas9 (1154-1368-VPR) fragment and pA017. The dCas9 (1154-1368-VPR) fragment was PCR amplified from pA046 (CAG_dCas9_VPR) vector using primer p880 and p812r.

pSA028 (CAG-NLS-InteinC-dCas9(204-1368)-VPR) was made in a Golden Gate reaction with dCas9(204-1368-VPR) fragment, InteinC fragment and pA017. The dCas9(204-1368-VPR) fragment was PCR amplified from pA046 (CAG_dCas9_VPR) vector using primer p873 and p812r.The InteinC fragment was from pSA015.

pSA029 (CAG-NLS-InteinC-dCas9(469-1368)-VPR) was made in a Golden Gate reaction with dCas9(469-1368-VPR) fragment, InteinC fragment and pA017. The dCas9(1-468) fragment was PCR amplified from pA046 (CAG_dCas9_VPR) vector using primer p879 and p812r. The InteinC fragment was from pSA015.

pSA030 (CAG-NLS-inteinC-dCas9(714-1368)-VPR) was made in a Golden Gate reaction with dCas9(714-1368-VPR) fragment, InteinC fragment and pA017. The dCas9(714-1368-VPR) fragment was PCR amplified from pA046 (CAG_dCas9_VPR) vector using primer p887 and p812r. The InteinC fragment was from pSA015.

pSA031 (CAG-NLS-InteinC-dCas9(1154-1368)-VPR) was made in a Golden Gate reaction with dCas9(1154-1368-VPR) fragment, InteinC fragment and pA017. The Cas9(1154-1368-VPR) fragment was PCR amplified from pA046 (CAG_dCas9_VPR) vector using primer p876 and p812r. The InteinC fragment was from pSA015.

pSA032 (CAG-NLS-dCas9(714-1153)-InteinN) was made in a Golden Gate reaction with dCas9(714-1153) fragment, InteinN fragment and pA017. The dCas9(714-1153) fragment was PCR amplified from pT096 (pENTR_NLS_dCas9) vector using primer p888 and p874.The InteinN fragment was from pSA005.

pSA033 (PENTR_NLS-dCas9-Krab) was made by inserting Krab fragment into pT096 (pENTR_NLS_dCas9) by using XbaI and XhoI. The Krab fragment was synthesized from Genewiz.

pSA034 (PENTR_NLS_LacZ_NLS_Krab) was made by inserting Krab fragment into pDS001 vector by using XbaI and XhoI.

pSA035 (CAG-NLS-dCas9-Krab) was prepared by combining pZ250 (pENTR_L4-CAG-L1) and pSA033 (PENTR-NLS-dCas9-Krab) with pZDonor_1-GTW-2 in a Gateway LR reaction.

pSA036 (PENTR_NLS_dCas9(714-1368)-Krab) was made in a Golden Gate

reaction with dCas9(714-1368) fragment and the pSA034 vector. The dCas9(714-1368) fragment was PCR amplified from the pT096 (pENTR_NLS_dCas9) vector using primer p887 and p3.

pSA037 (CAG-NLS-dCas9(714-1368)-Krab) was prepared by combining pZ250 (pENTR_L4-CAG-L1) and pSA036 (PENTR_NLS_dCas9(714-1368)-Krab) with pZDonor_1-GTW-2 in a Gateway LR reaction.

pSA038 (PENTR-NLS-LacZ-NLS-Suntag) was made by inserting Suntag fragment into pDS001 vector by using XbaI and XhoI. The Suntag fragment was PCR amplified from 1501 vector (donated by the Lei Qi Lab) using primer p4 and p5.

pSA039 (pENTR-NLS-inteinC-dCas9(1154-1368)-Suntag) was made in a Golden Gate reaction with inteinC fragment, dCas9(1154-1368) fragment and the pSA038 vector. The InteinC fragment was from the pSA015.The dCas9(1154-1368) fragment was PCR amplified from the pT096 (pENTR_NLS_dCas9) vector using primer p3 and p876.

pSA040 (CAG-NLS-ScFV-GB1-VP64-NLS) was made in a Golden Gate reaction with ScFV fragment, GB1 fragment, VP64 fragment and the pA017 vector. The ScFV fragment was PCR amplified from the 1504 plasmids (SCFV-SfGFP-VP64) (donated from the Lei Qi Lab) using primer pScFVF and pScFVR. The GB1 fragment was synthesized from Genewiz. The VP64 fragment was PCR amplified from the NMnCas9-VP64 (Addgene number 48676) using primer pVP64F and pVP64R.

pSA041 (CAG-NLS-ScFV-GB1-FKBP) was made in a Golden Gate reaction with ScFV fragment, GB1 fragment, FKBP fragment and the pA017 vector. The ScFV fragment was PCR amplified from the 1504 plasmids (SCFV-SfGFP-VP64) (donated from the Lei Qi Lab) using primer pScFVF and pScFVR. The GB1 fragment was synthesized from Genewiz. The FKBP fragment was synthesized from Genewiz.

pSA042 (CAG-NLS-FRB*-VP64) was made in a Golden Gate reaction with FRB* fragment, VP64 fragment and the pA017 vector. The FRB* was synthesized from Genewiz. The VP64 fragment was PCR amplified from the NMnCas9-VP64 (Addgene number 48676) using primer pVP64F and pVP64R.

pSA044 (PENTR-L1-NLS-LacZ-2A-TALE14-4xTarget^miR18a-L2) was made by inserting the LacZ fragment into the pWX45 (pENTR-L1-mkate2-T2A-TALE14-4xTarget^miR18a-L2)[1] by using MfeI and PspOM1.The LacZ fragment was PCR amplified from pDS001 using primer M13F and pLacZR.

pSA045 (pENTR-L1-NLS-LacZ-NLS-T2A-TALE14-4xTarget^FF4-L2) was made by inserting the LacZ fragment into the pWX22 (pENTR-L1-dTA-T2A-TALER14-4xTarget^FF4-L2)[1] by using MfeI and PspOMI. The LacZ fragment was PCR amplified

from pDS001 using primer M13F and pLacZR.

pSA046 (pENTR-L1-NLS-LacZ-NLS-T2A-TALE14--4xTarget^T19ab-L2) was made by inserting the LacZ fragment into the pWX49 (pENTR-L1-pENTR_L1_Kozak-mKate2-T2A-TALER14-4xT19ab_L2)[1] by using MfeI and PspOMI. The LacZ fragment was PCR amplified from the pDS001 using primer M13F and pLacZR.

pSA047 (pENTR-L1-NLS-LacZ-NLS-T2A-TALE14--4xTarget^T191-L2) was made by inserting the LacZ fragment into the pWX46 (pENTR_L1_Kozak-mKate2-T2A-TALER14-4xT191_L2)[1] by using MfeI and PspOMI. The LacZ fragment was PCR amplified from pDS001 using primer M13F and pLacZR.

pSA048 (pENTR-L1-NLS-LacZ-NLS-T2A-TALE9-4*Target^FF5-L2) was made by inserting the LacZ fragment into the pCH046 (pENTR_L1_Kozak-mKate2-T2A-TALE9-4xFF5_L2)[1] by using MfeI and PspOMI. The LacZ fragment was PCR amplified from pDS001 using primer M13F and pLacZR.

pSA049 (pENTR-L1-NLS-LacZ-NLS-T2A-TALE9-4*Target^miR21-L2) was made by inserting the LacZ fragment into the pCH153 (pENTR_L1_Kozak-mKate2-T2A-TALE9-4xmiR21_L2)[1] by using MfeI and PspOMI. The LacZ fragment was PCR amplified from pDS001 using primer M13F and pLacZR.

pSA050 (UASx5-T9+T9x3-NLS-LacZ-2A-TALE14-4xTarget^miR18a) was prepared by combining YQ5 (PENTR_L4-UASx5-T9+T9x3-R1)[1] and pSA044 (PENTR-L1-NLS-LacZ-2A-TALE14-4xTarget^miR18a-L2) with pT592(GTW) in a Gateway LR reaction.

pSA051 (UASx5-T9+T9x3-NLS-LacZ-2A-TALE14-4xTarget^FF4) was prepared by combining YQ5 (PENTR_L4-UASx5-T9+T9x3-R1) and pSA045 (PENTR-L1-NLS-LacZ-2A-TALE14-4xTarget^FF4-L2) with pT592(GTW) in a Gateway LR reaction.

pSA052 (UASx5-T9+T9x3-NLS-LacZ-2A-TALE14-4xTarget^T19ab) was prepared by combining YQ5 (PENTR_L4-UASx5-T9+T9x3-R1) and pSA046 (PENTR-L1-NLS-LacZ-2A-TALE14-4xTarget^T19ab-L2) with pT592(GTW) in a Gateway LR reaction.

pSA053 (UASx5-T9+T9x3-NLS-LacZ-2A-TALE14-4xTarget^T191) was prepared by combining YQ5 (PENTR_L4-UASx5-T9+T9x3-R1) and pSA047 (PENTR-L1-NLS-LacZ-2A-TALE14-4xTarget^T191-L2) with pT592(GTW) in a Gateway LR reaction.

pSA054 (UASx5-T14+T14x3-NLS-LacZ-2A-TALE9-4*Target^FF5) was

prepared by combining YQ8 (PENTR_L4-UASx5-T14+T14x3-R1)[1] and pSA048 (pENTR-L1-NLS-LacZ-T2A-TALE9-4*Target^FF5-L2) with pT592(GTW) in a Gateway LR reaction.

pSA055 (UASx5-T14+T14x3-NLS-LacZ-2A-TALE9-4*Target^miR21) was prepared by combining YQ8 (PENTR_L4-UASx5-T14+T14x3-R1) and pSA049 (pENTR-L1-NLS-LacZ-T2A-TALE9-4*Target^mirR21-L2) with pT592(GTW) in a Gateway LR reaction.

pSA056 (UASx5-T9+T9x3-NLS-InteinC-dCas9(714-1368)(VRER)-VPR-2A-TALE14-4xTarget^FF4) was made in a Golden Gate reaction with dCas9(714-1368)(VRER)-VPR fragment, InteinC fragment and pSA051 (UASx5-T9+T9x3-NLS-LacZ-2A-TALE14-4xTarget^FF4) vector. The dCas9(714-1368)(VRER)-VPR fragment was made by Golden Gate assembly to create the D1135V/G1218R/R1335E/T1337R mutant. The dCas9(714-1368)(VRER)-VPR sub-fragment1 was PCR amplified from pA046 using primer p13 and p887. The dCas9(714-1368)(VRER)-VPR subfragment2 was PCR amplified from pA046 using primer p12 and p14. The dCas9(714-1368)(VRER)-VPR subfragment3 was PCR amplified from pA046 using primer p15 and p16. The dCas9(714-1368)(VRER)-VPR subfragment3 was PCR amplified from the pA046 using primer p17 and p812r.

pSA057 (UASx5-T14+T14x3-NLS-InteinC-dCas9(714-1368)-Krab-2A-TALE9-4*Target^FF5) was made in a Golden Gate reaction with InteinC fragment, dCas9(714-1368)-Krab fragment and pSA054 (UASx5-T14+T14x3-NLS-LacZ-2A-TALE9-4*Target^FF5). The InteinC fragment was from pSA015.The dCas9(714-1368)-Krab was PCR amplified from pSA033 using primer p887 and pKrabR.

pSA058 (UASx5-T14+T14x3-NLS-InteinC-dCas9(714-1368)-Krab-2A-TALE9-4*Target^miR21) was made in a Golden Gate reaction with InteinC fragment, dCas9(714-1368)-Krab and pSA055 (UASx5-T14+T14x3-NLS-LacZ-2A-TALE9-4*Target^miR21). The InteinC fragment was from the pSA015. The dCas9(714-1368)-Krab was PCR amplified from pSA033 using primer p887 and pKrabR.

pSA059 (UASx5-T9+T9x3-NLS-InteinC-dCas9(714-1368)(VRER)-VPR-2A-TALE14-4xTarget^miR18a) was made in a Golden Gate reaction with InteinC fragment, dCas9(714-1368)(VRER)-VPR and pSA050 (UASx5-T9+T9x3-NLS-LacZ-2A-TALE14-4xTarget^miR18a). The InteinC fragment was from pSA015. The dCas9(714-1368)(VRER)-VPR fragment was PCR amplified from pSA056 (UASx5-T9+T9x3-NLS-InteinC-dCas9(714-1368)(VRER)-VPR-2A-TALE14-4xTarget^FF4) using primer p812r and p887.

pSA060 (UASx5-T14+T14x3-NLS-InteinC-dCas9(714-1368)(VRER)-VPR-2A-TALE9-4*Target^FF5) was made in a Golden Gate reaction with InteinC fragment, dCas9(714-1368)(VRER)-VPR and pSA054 (UASx5-T14+T14x3-NLS-LacZ-2A-

TALE9-4*Target^FF5) by Golden Gate Assembly. The InteinC fragment was from the pSA015. The dCas9(714-1368)(VRER)-VPR fragment was PCR amplified from the pSA056 (UASx5-T9+T9x3-NLS-InteinC-dCas9(714-1368)(VRER)-VPR-2A-TALE14-4xTarget^FF4) using primer p812r and p887.

pSA061 (UASx5-T9+T9x3-NLS-InteinC-dCas9(714-1368)-VPR-2A-TALE14-4xTarget^FF4) was made in a Golden Gate reaction with InteinC fragment, dCas9(714-1368)-VPR fragment and pSA051 (UASx5-T9+T9x3-NLS-LacZ-2A-TALE14-4xTarget^FF4). The InteinC fragment was from the pSA015. The dCas9(714-1368)-VPR was PCR amplified from the pA046 using primer p812r and p887.

pSA062 (UASx5-T9+T9x3-NLS-InteinC-dCas9(714-1368)-VPR-2A-TALE14-4xTarget^T19ab) was made in a Golden Gate reaction with InteinC fragment, dCas9(714-1368)-VPR fragment and pSA052 (UASx5-T9+T9x3-NLS-LacZ-2A-TALE14-4xTarget^T19ab). The InteinC fragment was from the pSA015. The dCas9(714-1368)-VPR was PCR amplified from the pA046 using primer p812r and p887.

pSA063 (UASx5-T9+T9x3-NLS-InteinC-dCas9(714-1368)-VPR-2A-TALE14-4xTarget^T191) was made in a Golden Gate reaction with InteinC fragment, dCas9(714-1368)-VPR fragment and pSA053(UASx5-T9+T9x3-NLS-LacZ-2A-TALE14-4xTarget^T191). The InteinC fragment was from the pSA015. The dCas9(714-1368)-VPR was PCR amplified from the pA046 using primer p812r and p887.

pSA064 (pENTR_modTRE1) was made by inserting a DNA fragment that contains 7 repeats of gRNAb binding site, the miniCMV and 3 repeats of gRNAb binding site into the pZ472 (L4_MCS_L1)[2] by using XhoI and SpeI. The DNA fragment was synthesized by Genewiz.

pSA065 (pModTRE1-EYFP) was prepared by combining pSA064 (pENTR_modTRE1) and pENTR_L1-EYFP-L2 [1] with pT592(GTW) in a Gateway LR reaction.

pSA066 (pModTRE2-mKate2) was prepared by combining pSA067 (pENTR_modTRE2) and pZ454 (pENTR_L1-mKate_L2) [1] with pT592(GTW) in a Gateway LR reaction.

pSA067 (pENTR_modTRE2) was made by inserting a DNA fragment that contains 7 repeats of gRNAb and the miniCMV into the pZ472 (L4_MCS_L1) by using XhoI and SpeI. The DNA fragment was synthesized by Genewiz.

pSA068 (pModTRE2-mKate2-4*Target^FF5) was prepared by combining pSA067 (pENTR_modTRE2) and pZ394 (pENTR-L1-mKate2-4*Target^FF5-L2)[2] with

pT592(GTW) in a Gateway LR reaction.

pSA069 (pTRE-EYFP-4*Target^FF4) was made by inserting 4 repeats of shRNA-FF4 target sites into pB044 (pTRE-EYFP) by using BsaI and XbaI.

pSA070 (CAG-NLS-dCas9(1-1153)(D1135E)-InteinN) was made in a Golden Gate reaction with dCas9(1-1153)(D1135E) fragment and pSA005 (the donor vector for InteinN) and pA017. The dCas9(1-1153)(D1135E) fragment was PCR amplified from pSA077 (CAG-NLS-dCas9(D1135E/R1335Q/T1337R)) using primer p870 and p874.

pSA071 (CAG-NLS-InteinC-dCas9(1154-1368)(R1335Q/T1337R)-VPR) was made in a Golden Gate reaction with dCas9(1154-1368)(R1335Q/T1337R) fragment and pSA015 (the donor vector for InteinC) and pA017. The dCas9(1154-1368)(R1335Q/T1337R)-VPR fragment was PCR amplified from pSA077 (CAG-NLS-dCas9(D1135E/R1335Q/T1337R)) using primer p812r and 876.

pSA072 (CAG-NLS-InteinC-dCas9(1154-1368)-VP64) was made in a Golden Gate reaction with dCas9(1154-1368)-VP64 fragment, pSA015 (the donor vector for InteinC) and pA017. The dCas9(1154-1368) fragment was PCR amplified from pA046 vector using primer p876 and p18.

pSA073 (CAG-NLS-InteinC-Cas9(204-1368)) was made in a Golden Gate reaction with Cas9(204-1368) fragment, pSA015 (the donor vector for InteinC) and pA017. The Cas9(204-1368) fragment was PCR amplified from pT106 (CAG_cas9) vector using primer p873 and p3.

pSA074 (CAG-NLS-InteinC-Cas9(469-1368)) was made in a Golden Gate reaction with Cas9(469-1368) fragment, pSA015 (the donor vector for InteinC) and pA017. The Cas9(469-1368) fragment was PCR amplified from pT106 (CAG_cas9) vector using primer p879 and p3.

pSA075 (CAG-NLS-InteinC-Cas9(714-1368)) was made in a Golden Gate reaction with Cas9(714-1368) fragment, pSA005 (the donor vector for InteinN) and pA017. The Cas9(714-1368) fragment was PCR amplified from pT106 (CAG_cas9) vector using primer p887 and p3.

pSA076 (CAG-NLS-InteinC-Cas9(1154-1368)) was made in a Golden Gate reaction with Cas9(1154-1368) fragment, pSA005 (the donor vector for InteinN) and pA017. The Cas9(1154-1368) fragment was PCR amplified from pT106 (CAG_cas9) vector using primer p876 and p3.

pSA077 (CAG-NLS-dCas9(D1135E/R1335Q/T1337R)-VPR) was made in a Golden Gate reaction with dCas9(D1135E/R1335Q/T1337R) subfragment1, dCas9 (D1135E/R1335Q/T1337R) subfragment2 and dCas9 (D1135E/R1335Q/T1337R)

subfragment3 and pA017. The dCas9(D1135E/R1335Q/T1337R) subfragment1 was PCR amplified from pA046 using primer p870 and p701. The dCas9 (D1135E/R1335Q/T1337R) subfragment2 was PCR amplified from pA046 using primer p702 and p703. The dCas9 (D1135E/R1335Q/T1337R) subfragment3 was PCR amplified from pA046 using primer p704 and p812r.

## Supplementary References


1. Li, Y. et al. Modular construction of mammalian gene circuits using TALE transcriptional repressors. Nat. Chem. Biol. 1–28 (2015).

2. Xie, Z., Wroblewska, L., Prochazka, L., Weiss, R. & Benenson, Y. Multi-input RNAi-based logic circuit for identification of specific cancer cells. *Science* **333,** 1307–1311 (2011).
